# A complex glenoid fracture following high-energy trauma: Case report and literature review

**DOI:** 10.1097/MD.0000000000043466

**Published:** 2025-08-08

**Authors:** Ștefan-Dragoș Tîrnovanu, Awad Dmour, Bogdan Puha, Dragoș-Cristian Popescu, Vasile Lisnic, Alexandru Filip, Corina Ciupilan, Ovidiu Alexa

**Affiliations:** a Department of Orthopaedics and Traumatology, University of Medicine and Pharmacy “Grigore T. Popa” Iasi, Iasi, Romania; b Department of Orthopaedics and Traumatology, “Sf. Spiridon” Emergency Hospital Iasi, Iasi, Romania.

**Keywords:** glenoid fossa, Ideberg, motorcycle accident, scapula

## Abstract

**Rationale:**

Glenoid fractures, representing approximately 10% of scapular fractures, are uncommon but clinically significant due to their frequent association with high-energy trauma. Without timely and adequate management, such injuries may lead to complications including nonunion, osteoarthritis, and chronic instability, especially when fracture patterns are complex or extend into the scapular body. This report highlights the unique diagnostic and therapeutic challenges of managing an Ideberg type Vb glenoid fracture.

**Patient concerns:**

A 32-year-old male motorcyclist presented with significant swelling and ecchymosis around the left shoulder and axilla, accompanied by complete functional impairment of the shoulder joint. Despite the severity of the trauma, neurological sensitivity was preserved on admission.

**Diagnoses:**

Imaging through radiography and computed tomography with 3-dimensional reconstruction revealed a comminuted glenoid cavity fracture, displacement of the coracoid process, and extension of the fracture line into the scapular body – classified as an Ideberg type Vb fracture.

**Interventions:**

Surgical management was conducted via the Judet approach, involving open reduction and internal fixation using 2 reconstruction plates. These were precontoured on a cadaver scapula model and intraoperatively adapted to the patient’s anatomy. Postoperative immobilization with a Velpeau bandage was maintained for 30 days. At 6 months, a secondary procedure – neurotization using sural nerve grafts and direct neuromuscular neurorrhaphy via the Giorgio Brunelli technique – was performed due to persistent motor deficits attributed to infraspinatus nerve injury.

**Outcomes:**

Initial postoperative follow-up showed persistent deficits in external rotation and abduction. Following neurotization, the patient exhibited gradual and substantial functional recovery, with a Constant shoulder score of 86 documented at 2 years post-trauma.

**Lessons:**

This case emphasizes the complexity of Ideberg type Vb glenoid fractures and the necessity for comprehensive diagnostic imaging, individualized surgical planning, and interdisciplinary collaboration. Preoperative plate contouring with cadaveric models and targeted neurological reconstruction were pivotal in achieving a favorable long-term outcome.

## 1. Introduction

Scapular fractures, particularly those involving the glenoid, are rare and associated with high-energy trauma, often resulting from incidents such as vehicle collisions or falls from significant heights.^[1]^ Accounting for <1% of all fractures,^[2]^ these injuries typically present in complex patterns, frequently leading to intra-articular damage, and require careful assessment to determine the optimal treatment pathways. Given the critical role of the scapula in shoulder function and stability, fractures involving the scapula, especially those with glenoid involvement, can lead to significant morbidity if not accurately diagnosed and managed.^[3]^ The Ideberg classification is commonly employed to categorize these fractures based on the involvement of various parts of the glenoid and scapular body, which helps guide clinical decision making.^[4]^

Motorcycle accidents, a leading cause of high-energy trauma, disproportionately affect young male adults and are associated with severe injuries, including traumatic brain injuries and fractures of the upper and lower limbs.^[5]^ The propensity for more severe outcomes in these incidents is heightened by factors such as exposure to high speeds, limited protection, and often inadequate use of safety equipment beyond helmets. In such cases, the likelihood of complex fractures such as those involving the scapula is high, particularly when the impact results in forceful contact with the ground or other vehicles.

This report details the case of a patient who suffered a displaced glenoid fracture resulting from a motorcycle accident, illustrating the challenges in the diagnosis, classification, and treatment of scapular injuries in high-energy trauma contexts.

## 2. Case presentation

We report a case of complex glenoid fracture and describe the clinical findings, radiological appearances, management challenges, and clinical outcomes. Written informed consent was obtained from the patient for participation in this study, including the use of anonymized data and clinical images for publication. The Ethical Board of the Emergency Hospital “Sf. Spiridon” Iasi was obtained according to international regulations.

**Figure 1.**
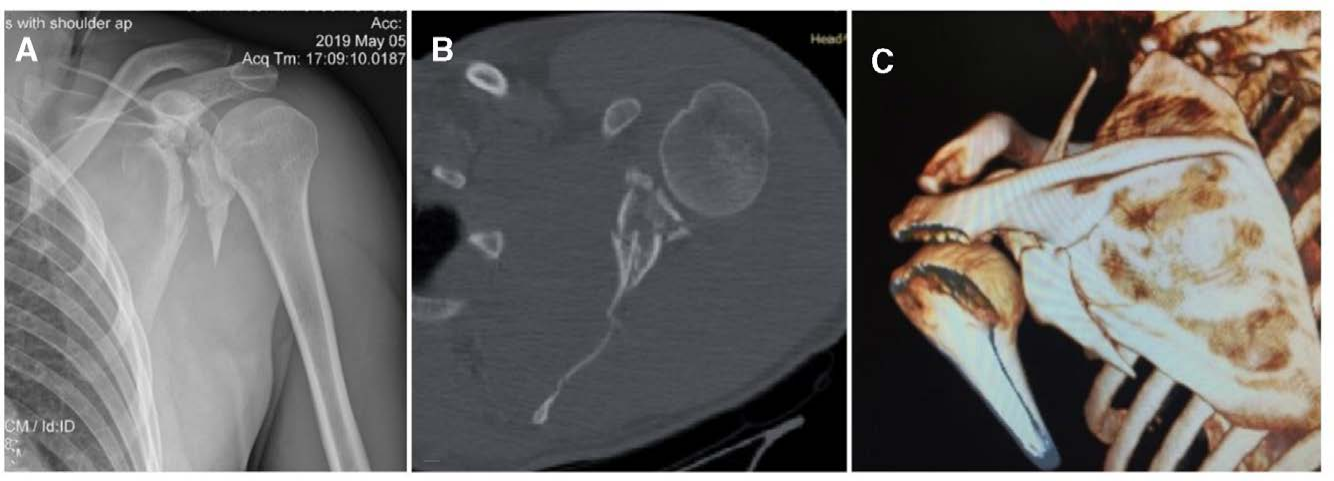
(A) Anteroposterior X-ray of the left shoulder, (B) CT axial view, (C) 3D CT reconstruction.

A 32-year-old man was brought to the emergency department following a motorcycle accident. Physical examination revealed left periscapular edema and bruising, and the patient was unable to actively abduct, internally or externally rotate the shoulder, or resist the passive performance of these movements. The ulnar and radial pulses were palpable and symmetric, respectively. Sensation for light touch was intact throughout the deltoid, forearm, and hands. Plain radiography revealed a fracture at the level of the glenoid and surgical neck of the left scapula (Fig. 1A), prompting a CT examination. The CT revealed a complex scapular fracture with detachment of the coracoid process, comminution of the glenoid cavity, diastasis relative to the scapular body, and a fracture line at the body level extending immediately to the infraspinatus with displacement at the fracture site, indicating an Ideberg-type Vb fracture (Fig. 1B and C).

**Figure 2.**
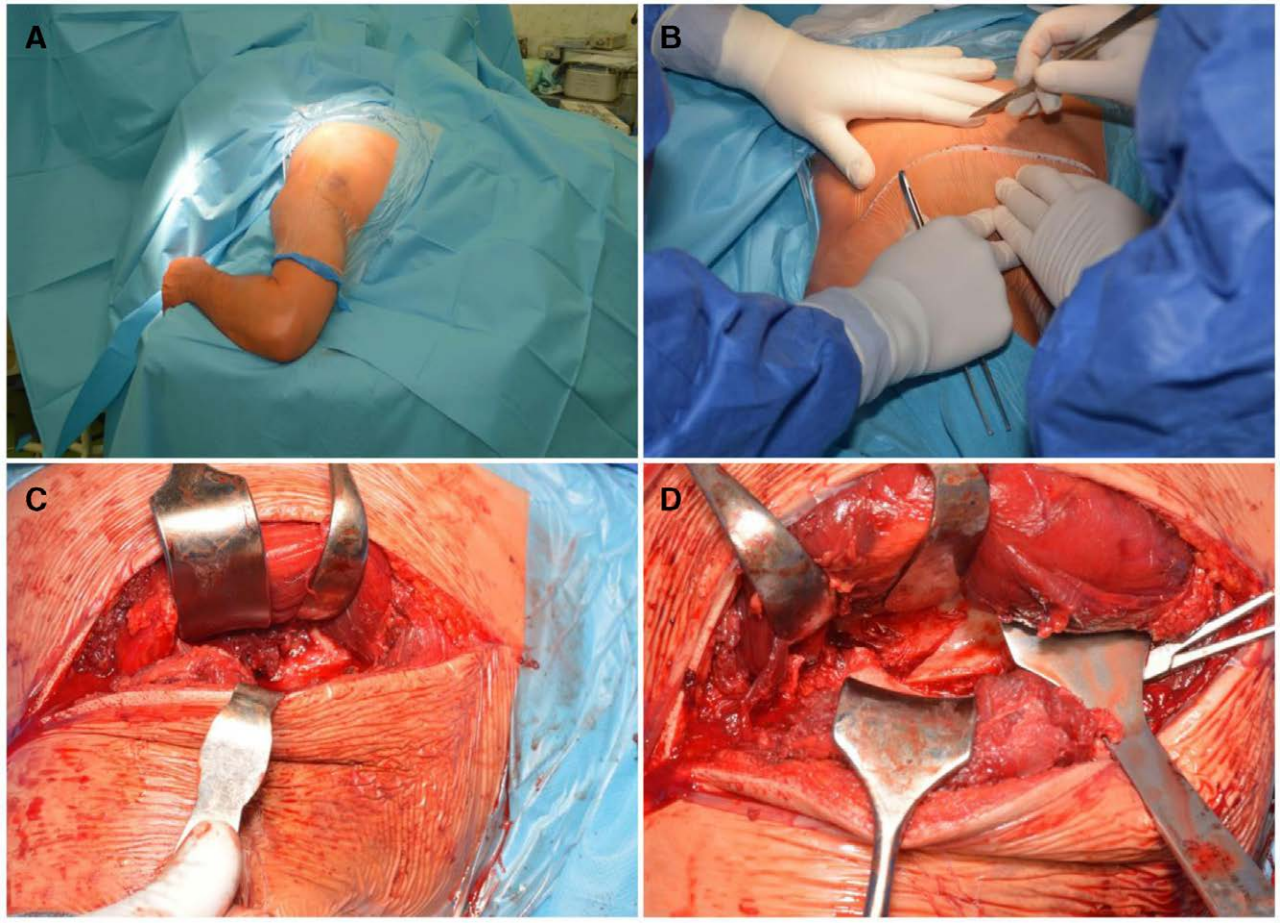
(A) Patient in prone position after general anesthesia, (B) modified posterior Judet approach, (C) the deltoid is dissected near its insertion at the level of the scapular spine and acromion, (D) the interval between the infraspinatus muscle and the teres minor muscle an visualization of the later edge of the scapula.

The patient underwent surgery using a modified Judet approach, with the patient in the prone position after the induction of general anesthesia. The incision started at the upper surface of the acromion tip and was directed toward the inferior angle of the scapula, parallel to the lateral edge of the scapula. The deltoid muscle was identified and dissected near its insertion point, at the level of the scapular spine and acromion. It is retracted laterally, with care taken to protect the axillary nerve and posterior circumflex humeral artery. The interval between the infraspinatus and teres minor muscles was identified to visualize the lateral edge of the scapula and posterior portion of the joint capsule (Fig. 2).

Before surgery, to optimize surgical efficiency and accuracy, the reconstruction plates were pre-contoured preoperatively using a cadaveric scapula model, shaped according to the patient’s specific fracture morphology as analyzed from preoperative CT scans. This preparatory step was intended to minimize intraoperative time and ensure precise anatomical conformity during fixation.

**Figure 3.**
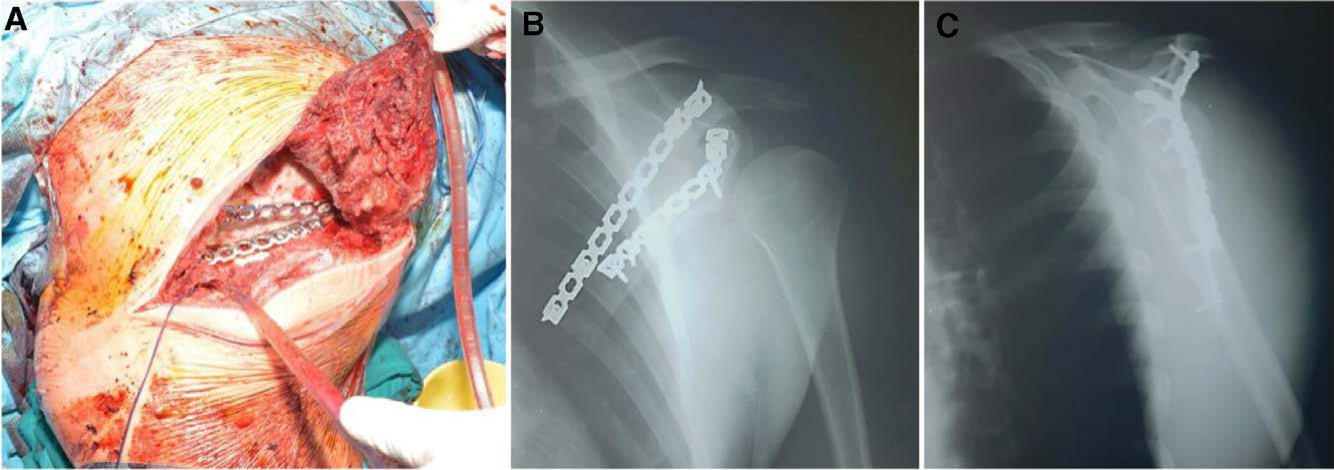
(A) Intraoperative aspect of plate positioning, postoperative X-ray (anteroposterior view – B) of the shoulder and lateral view (C).

Open reduction and internal fixation was performed, 1 reconstruction plate was initially fixed to the glenoid cavity with 2 screws, ensuring that they did not penetrate the joint. Fracture reduction was verified under fluoroscopic control, and the plate was fixed to the lateral edge of the scapula using 3 screws. After securing the scapular neck, a second reconstruction plate was used to reduce the fracture at scapular body level. This was fixed to the acromion with 2 screws and to the scapular body below with the other 2 screws. Postoperative control radiography showed good positioning of the osteosynthesis material and proper fracture reduction (Fig. 3).

## 3. Results

### 3.1. Immediate postoperative period

Intraoperative fluroscopy confirmed proper reduction and implant placement. The upper limb was immobilized using a shoulder brace for 4 weeks. Physiotherapy was initiated with passive mobilization and pendulum movements in the first 2 weeks, followed by active mobilization at 4 to 6 weeks postoperatively.

### 3.2. Two months follow-up

The patient exhibited limited active shoulder motion, with flexion of 60°, abduction of 35°, and extension of 45°. The Constant Score was 28, indicating a poor early functional outcome. A marked deficit in external rotation and abduction raised clinical suspicion of infraspinatus muscle paresis, likely due to suprascapular nerve injury, prompting further multidisciplinary evaluation.

### 3.3. Six-month follow-up and revision surgery

Surgical exploration was undertaken. The osteosynthesis hardware was removed by the orthopedics team through an approach along the previous postoperative scar. A nerve graft, approximately 26 cm long, represented by the sural nerve, was harvested from the left lower limb. A branch of the infraspinatus muscle originating from the suprascapular nerve was identified. The nerve was interrupted, with a defect of approximately 10 cm embedded in fibrous scar tissue. Neuroneuronal neurotization was performed with 2 sural nerve cables between the proximal end of the nerve and the distal branches, just before entering the muscle body, and direct neuromuscular neurorrhaphy using the Brunelli technique.

### 3.4. At the 2-year follow-up

**Figure 4.**
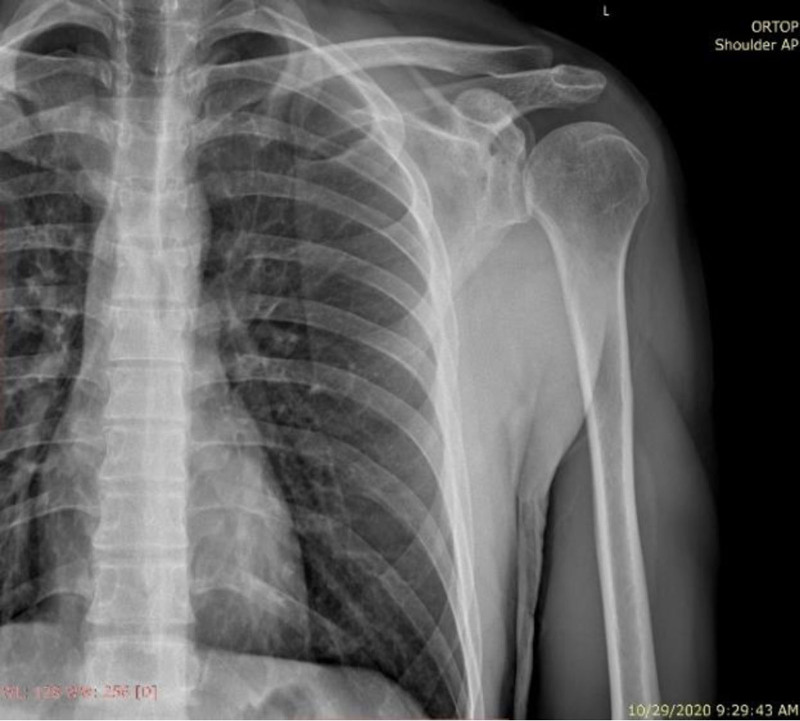
Anteroposterior X-ray at 2-year follow-up.

**Figure 5.**
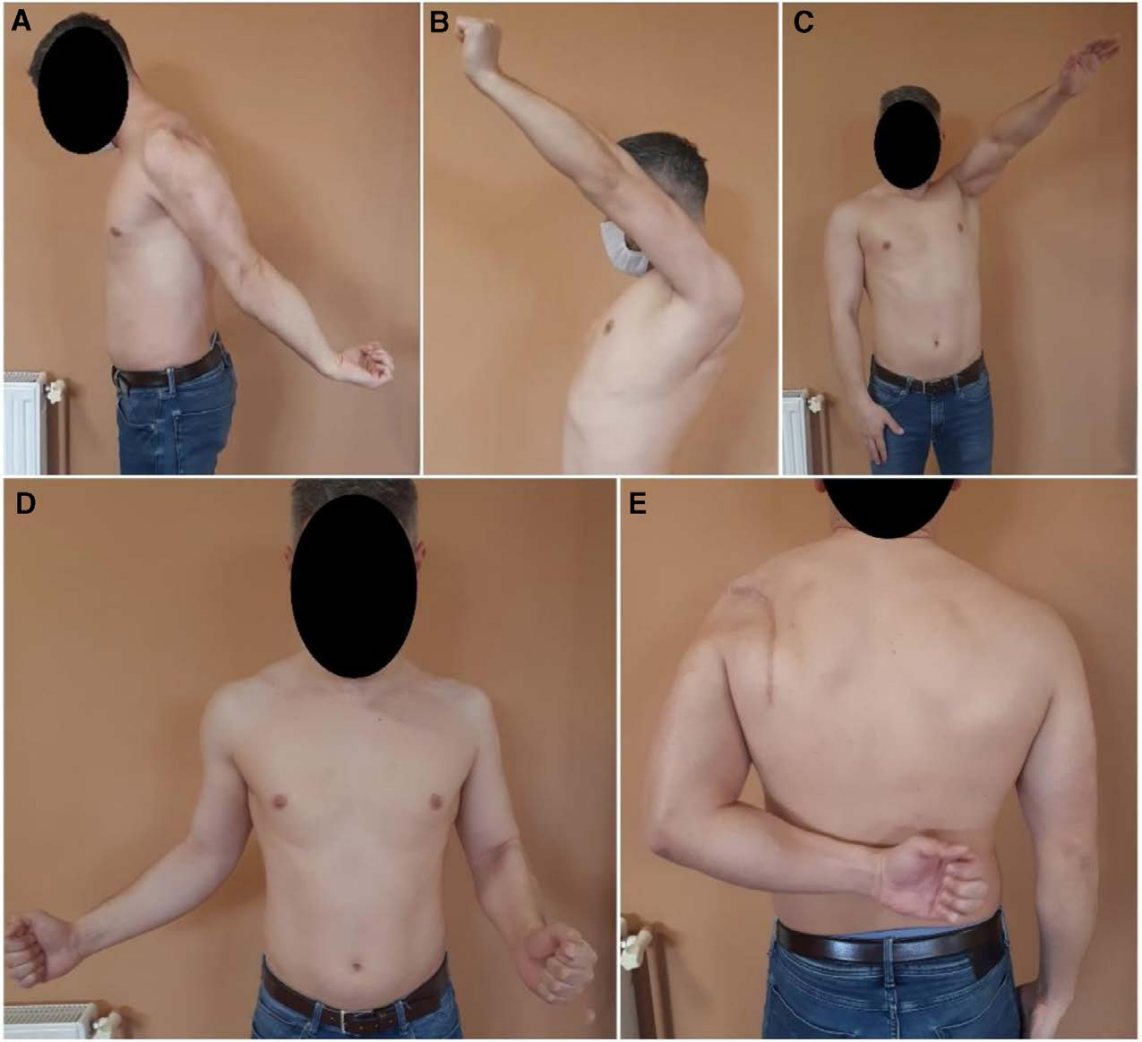
(A and E) Backward extension and internal rotation of the left shoulder within normal limits, (B) flexion up to 130°, (C) abduction reaching 130°, (D) limited extern rotation, only 15°.

Radiographic evaluation at 2 years confirmed complete fracture consolidation without signs of malunion or joint degeneration (Fig. 4). Clinically, the patient achieved active flexion of 130° and abduction of 130° (Fig. 5). Internal rotation and backward extension were within normal limits. External rotation remained limited to 15°. The Constant Score had improved to 86, reflecting a good functional outcome despite residual weakness in external rotation.

The Constant Score improved from 28 (poor function) at 2 months to 86 (good function) at 2 years, indicating substantial recovery of shoulder function following both ORIF and neurotization.

Radiographic follow-up at 2 years confirmed maintained fracture reduction and absence of degenerative changes. Clinically, although range of motion recovered significantly, the patient exhibited persistent weakness in external rotation – consistent with partial but incomplete nerve regeneration.

## 4. Discussion

This case underscores the complexities involved in managing a rare Ideberg-type Vb glenoid fracture with associated neurological injury. The literature confirms that glenoid fractures are infrequent, comprising <1% of fractures, and pose significant clinical challenges owing to their potential impact on shoulder stability and motion. Studies have emphasized that high-energy trauma, such as that sustained in motor vehicle accidents, often leads to severe injuries with complex presentations.

Optimal outcomes in complex glenoid fractures require ORIF, particularly in cases of significant displacement or comminution. The modified Judet approach, as applied in this case, offers excellent access to the posterior scapula, allowing precise anatomical restoration of the glenoid.^[6]^ The literature also supports the use of pre-contoured plates based on 3D CT reconstructions, as this technique aids in achieving stable fixation and reducing operative time. Cole et al and Gauger et al demonstrated that 3D imaging-based surgical planning can significantly improve the alignment accuracy and outcomes in shoulder fractures, which is particularly beneficial for glenoid fractures with extensive comminution.^[7,8]^

The patient’s persistent deficits in external rotation and abduction, attributed to an infraspinatus nerve injury, align with findings in cases where nerve involvement complicates shoulder trauma. Nerve damage in glenoid fractures is less frequently documented, but can arise from direct injury or secondary compression, leading to functional impairment even after successful skeletal stabilization. Studies, such as those by Tatro et al, highlight that neurovascular complications in scapular fractures may prolong recovery and often necessitate specialized interventions, including neurotization, when conservative measures prove ineffective.^[3]^ In this case, the use of sural nerve grafts for neurotization reflects a growing body of evidence supporting this approach for restoring partial nerve function in complex fractures with nerve involvement.

Rehabilitation after ORIF and neurotization is crucial for functional outcomes, as early physical therapy can prevent joint stiffness, promote muscle recovery, and support nerve regeneration. Yokoyama et al research on postoperative rehabilitation in complex elbow and shoulder fractures emphasizes early guided mobilization to restore range of motion and reduce long-term disability.^[9]^ In the present case, a phased rehabilitation protocol tailored to the patient’s nerve injury played an essential role in the functional recovery, although complete restoration remains challenging.

Outcomes in glenoid fractures with neurological impairment are variable and heavily influenced by injury severity, nerve involvement, and the precision of surgical intervention. Literature on similar cases suggests that multidisciplinary management, incorporating precise surgical fixation and nerve repair, is vital for maximizing recovery potential. Studies by Limb et al and Solomon et al reinforced that comprehensive patient-specific strategies yield better long-term outcomes in complex shoulder injuries, although full recovery may still be limited by the extent of initial nerve damage.^[6,10]^

## 5. Limitations

This case report presents several limitations inherent to its design. Firstly, as a single-patient case study, the generalizability of the findings is inherently restricted. Secondly, although advanced imaging and individualized preoperative planning were employed, the long-term prognosis regarding full neurological recovery remains uncertain due to the complexity of nerve injuries. Lastly, while the use of cadaver models and individualized surgical approaches represents an emerging technique, comparative studies are required to validate their superiority over conventional methods.

## 6. Conclusion

This case exemplifies the intricate challenges associated with high-energy glenoid fractures with concurrent nerve injury. The modified Judet approach and personalized neurotization strategy facilitated significant recovery, underscoring the importance of precise surgical techniques and collaborative specialized care. Although the outcomes were positive, this case also highlights the ongoing need for advancements in nerve repair and postoperative rehabilitation to further improve functional recovery in similar complex fractures.

## Author contributions

**Conceptualization:** Ștefan-Dragoș Tîrnovanu, Bogdan Puha, Ovidiu Alexa.

**Data curation:** Ștefan-Dragoș Tîrnovanu, Alexandru Filip.

**Formal analysis:** Corina Ciupilan.

**Investigation:** Awad Dmour, Dragoș-Cristian Popescu, Corina Ciupilan.

**Methodology:** Awad Dmour, Dragoș-Cristian Popescu, Vasile Lisnic, Alexandru Filip.

**Project administration:** Alexandru Filip.

**Resources:** Vasile Lisnic, Alexandru Filip.

**Supervision:** Awad Dmour, Dragoș-Cristian Popescu, Vasile Lisnic.

**Validation:** Dragoș-Cristian Popescu.

**Visualization:** Ovidiu Alexa.

**Writing – original draft:** Ștefan-Dragoș Tîrnovanu, Bogdan Puha, Ovidiu Alexa.

**Writing – review & editing:** Ștefan-Dragoș Tîrnovanu, Awad Dmour, Bogdan Puha, Corina Ciupilan, Ovidiu Alexa.
